# Matching medical staff to long term care facilities to respond to COVID-19 outbreak

**DOI:** 10.1186/s12913-023-09594-2

**Published:** 2023-06-07

**Authors:** Hamid Reza Zarei, Mahsa Ghanbarpour Mamaghani, Ozlem Ergun, Patricia Yu, Leanne Winchester, Elizabeth Chen

**Affiliations:** 1 Department of Mechanical & Industrial Engineering, Northeastern University, Boston, MA USA; 2 Executive Office of Health and Human Services, Boston, MA USA; 3 Graduate School of Nursing, University of MA Chan Medical School – Commonwealth Medicine, Worcester, MA USA; 4 Executive Office of Elder Affairs, Boston, MA USA

**Keywords:** COVID-19, Long term care facilities, Staff shortages, Resource allocation, Optimal assignment

## Abstract

**Background:**

Staff shortage is a long-standing issue in long term care facilities (LTCFs) that worsened with the COVID-19 outbreak. Different states in the US have employed various tools to alleviate this issue in LTCFs. We describe the actions taken by the Commonwealth of Massachusetts to assist LTCFs in addressing the staff shortage issue and their outcomes. Therefore, the main question of this study is how to create a central mechanism to allocate severely limited medical staff to healthcare centers during emergencies.

**Methods:**

For the Commonwealth of Massachusetts, we developed a mathematical programming model to match severely limited available staff with LTCF demand requests submitted through a designed portal. To find feasible matches and prioritize facility needs, we incorporated restrictions and preferences for both sides. For staff, we considered maximum mileage they are willing to travel, available by date, and short- or long-term work preferences. For LTCFs, we considered their demand quantities for different positions and the level of urgency for their demand. As a secondary goal of this study, by using the feedback entries data received from the LTCFs on their matches, we developed statistical models to determine the most salient features that induced the LTCFs to submit feedback.

**Results:**

We used the developed portal to complete about 150 matching sessions in 14 months to match staff to LTCFs in Massachusetts. LTCFs provided feedback for 2,542 matches including 2,064 intentions to hire the matched staff during this time. Further analysis indicated that nursing homes and facilities that entered higher levels of demand to the portal were more likely to provide feedback on the matches and facilities that were prioritized in the matching process due to whole facility testing or low staffing levels were less likely to do so. On the staffing side, matches that involved more experienced staff and staff who can work afternoons, evenings, and overnight were more likely to generate feedback from the facility that they were matched to.

**Conclusion:**

Developing a central matching framework to match medical staff to LTCFs at the time of a public health emergency could be an efficient tool for responding to staffing shortages. Such central approaches that help allocate a severely limited resource efficiently during a public emergency can be developed and used for different resource types, as well as provide crucial demand and supply information in different regions and/or demographics.

## Background

Throughout the COVID-19 outbreak, healthcare providers have suffered extended medical product and staff shortages [1]. To reduce the impact of these shortages a wide variety of measures were taken, such as bringing medical staff from all over the country to the epicenter at Hubei, China, and using students as temporary medical staff in Denmark [1, 2]. Long Term Care Facilities (LTCFs), where one of the most vulnerable population segments resides, suffered some of the worst shortages [3-5]. Based on the Centers for Medicare and Medicaid Services (Data.CMS.gov), roughly 20% of nursing homes (NHs) in the US have faced severe personal protective equipment (PPE) and staff shortages during the COVID-19 outbreak [6, 7]. As a result, the pandemic impacted LTCF residents more severely than most other population segments. Similar situations in LTCFs were reported in other countries such as Spain, England, and Italy [8-10].

The effect of LTCF staff shortages on quality of care, staff's work burden, and the likelihood of infection among residents during the COVID-19 pandemic is emphasized by prior studies [3, 4, 7, 11, 12]. In the US, the first LTCF COVID-19 infection case was reported in King County, Washington, on Feb 28th, 2020 [13]. From this date until Mar 28th, 2020, 30 LTCFs with confirmed cases were identified in King County [13]. One reason for such a quick spread of COVID-19 among LTCFs was identified as the practice of sharing medical staff, which led to LTCFs requiring staff not to work in multiple facilities [13, 14]. This restriction together with other pandemic related hardships including, staff's unwillingness or inability to work in COVID-19 positive settings and lack of childcare, resulted in LTCFs facing insurmountable challenges to satisfy their staffing needs at minimal levels. These staff shortages in turn resulted in negative outcomes even including forced evacuation of residents in some cases [3]. Moreover, several studies showed that the risk of COVID-19 infection doubled in an LTCF if the facility has fewer Registered Nurses (RNs) than the minimum recommended number [4]. Therefore, finding approaches to alleviate LTCF staff shortages has been one of the critical challenges for policymakers in responding to COVID-19 outbreak.

To relieve LTCF staff shortages, various approaches were implemented by different states within the US. For instance, in some cases, staff with suspected or confirmed COVID-19 could work in an LTCF under CDC guidelines [15, 16]. Other response initiatives included extending the temporary licensing of medical staff that do not have an active license, rapid training, engaging National Guard, using volunteer workers and students, providing a list of candidates to facilities tailored to their staffing needs, launching educational portals, and developing hiring and job posting portals [17].

Many studies point out the importance of having an adequate level of medical staff in LTCFs. Literature, covering several different fields including operations management, health systems etc., has a significant number of papers focus on matching resources to demand [18], and a branch of them is devoted to the healthcare systems, including nurse scheduling [19-21], patient allocation [22], and allocation of medical resources [23, 24]. However, these rarely suggest a central and effective way of dynamically matching medical staff to facilities in a public health emergency with severely limited resources, which is the central goal of this study.

To assist LTCFs in Massachusetts (MA), Commonwealth's Executive Office of Elder Affairs (EOEA) brought together subject matter experts from the Executive Office of Health and Human Services (EOHHS), Northeastern University, and University of Massachusetts Chan Medical School to design and operate a web portal to centrally recruit and allocate medical staff to LTCFs experiencing acute vacancies. The portal served as a central repository for collecting medical staff demand and supply data and designing a framework to match the supply to LTCFs' demand centrally and efficiently. The developed portal, a website that intakes data from medical staff and LTCFs, and the matching framework for allocating medical staff supply to LTCFs’ demand will be described in the following sections. We also developed statistical models to determine the most salient features that induced the LTCFs to submit feedback on their matches to the portal.

## Methods

### Portal overview

**Fig. 1:**
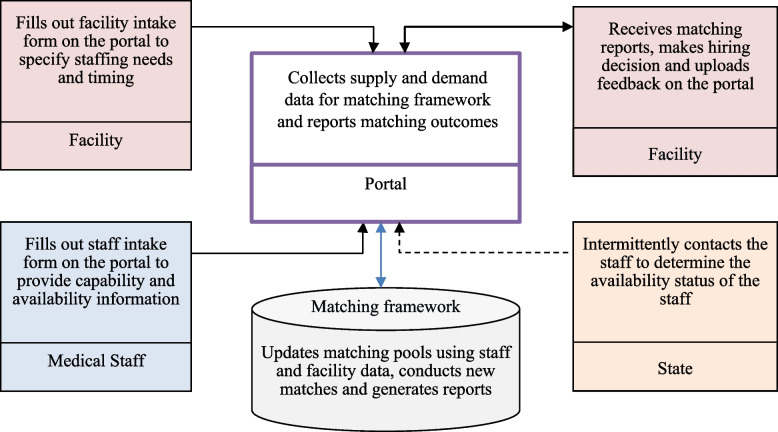
Overview of the structure of the matching framework

Medical staff and facilities put their information and needs in the portal by filling intake forms. Then, by using the information collected, the matching framework creates one pool of available medical staff and another pool for eligible facility demand for each round of allocation and matches medical staff to facilities considering various restrictions and policies. Based on the matching results, matching reports, which include the information of the matched staff, are created and sent to the facilities through the portal. Afterward, facilities contact the staff and were asked to provide feedback to the portal by entering whether they are intending to hire or did not hire the staff. In the matching framework context, facilities intending to hire staff meant that they decided to move forward with the matched staff to the next step of the hiring process, such as an interview or a job offer. On the other hand, a did not hire response meant that facilities considered the matched staff but decided to not proceed to the next step of the hiring process, which could have been for a variety of reasons that were not necessarily reported on the portal. As the portal evolved, added functionality enabled facilities to continuously update the status of the matched staff based on their interactions. In addition to the facilities, the state contacts the medical staff occasionally and updates the portal with their hiring status. Figure 1 shows an overview of the portal activities.

### Creating the portal and gathering data

The Massachusetts COVID-19 long-term care facility staffing portal intakes facility data has 43 features, including contact information, location, public transport accessibility, number of needed staff per position, and need-by date. The medical staff intake form has questions regarding 57 features, including personal and professional information, date of availability, and transportation preference.

**Fig. 2:**
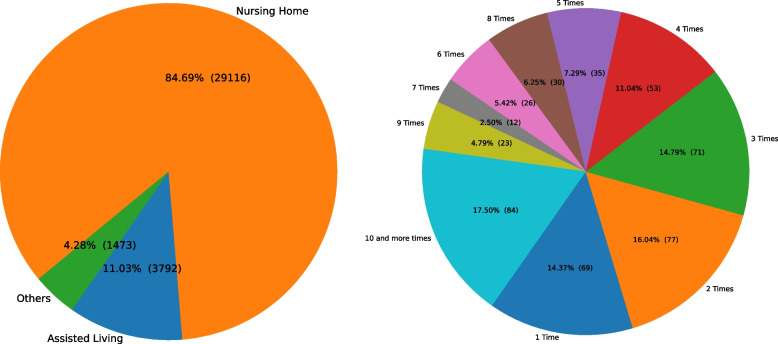
Distribution of demand among different facility types (left) and distribution of number of demand records facilities entered (right)

The portal went live on April 08, 2020, and the first matching was conducted two days later. Between April 8^th^, 2020, and June 29^th^, 2021, when the portal was operational, 5,398 medical staff registered through the portal. Furthermore, after removing some outlier facilities that constantly entered demand, 480 unique facilities (around 77.17% of total LTCFs in MA) voluntarily entered demand for 34,381 medical staff for ten different job types. Different types of facilities, including NHs, Assisted Living (AL), and Rest Homes, have used the portal. Figure 2 (left) indicates that NHs have entered significantly more demand than other types of LTCFs and (right) that 85.63% (411 out of 480) of the facilities that used the portal entered demand more than once. The EOEA and portal operations team encouraged medical staff to register through the portal by supporting the creation of temporary information campaigns for informing medical staff about the existence of the matching framework. In addition, for a limited time, MA government offered $1000 bonus to medical staff who get hired by an LTCF and work there for a certain time.^1^

**Fig. 3:**
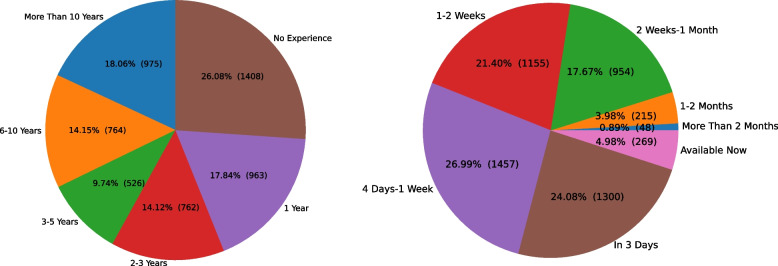
Distribution of medical staff’s years of experience (left) and distribution of medical staff’s availability from the time they enter the portal (right)

**Fig. 4:**
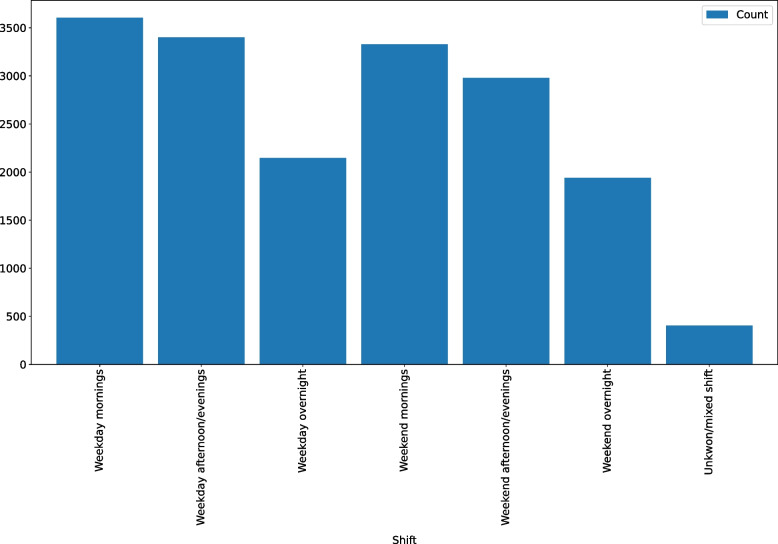
Availability distribution of registered medical staff for different shifts

Moreover, Fig. 3 (left) shows that 43.92% (2,371 out of 5,398) of the medical staff that used the portal have one year or no experience. Figure 3 (right) presents the distribution of medical staff’s availability from the time they enter the portal, where 77.45% (4,181 out of 5,398) of the medical staff are ready to work within two weeks of filling intake forms. Figure 4 indicates that the number of staff available during overnight shifts, both weekday and weekend, is lower than all other shifts.

Table 1 shows the full name and abbreviations of positions, the total demand entered by facilities for the given position, and the total number of staff that entered the portal for each position. As shown in the same table, except for RCAs,^2^ the demand is more than the supply for all positions.

**Table 1:** Different positions and total supply/demand for each position

Position title	Abbreviation	Demand for the position	Number of registered staff for the position
Activities coordinator	AC	1046	210
Certified Nursing Assistant	CAN	17,320	1,221
Licensed Independent Social Worker	LICSW	383	42
Licensed Practical Nurse	LPN	7,670	518
Occupational Therapist	OT	436	57
Occupational Therapist Assistant	OTA	170	29
Physical Therapist	PT	185	83
Physical Therapist Assistant	PTA	170	36
Registered Nurse	RN	7,001	1,306
Resident Care Assistant	RCA	1,795	1,896

### Matching model

We developed a mathematical programming model to match medical staff efficiently and optimally given an objective to facilities. Mathematical programming uses mathematical expressions to describe a system and a process and optimize this process with respect to a defined objective. Mathematical programming has been applied in various matching problems, including healthcare settings [19-27].

The developed mathematical model matches supply to demand based on a set of criteria, including position type, needed skills or licenses for staff, maximum distance to travel, public transportation accessibility, employment preferences (short-term/long-term), staff availability, and facility demand need-by dates. To match staff to facilities, we defined a matching weight for each staff-facility pair. Matching weights were designed to reflect the desirability of a match and included a function of distance between staff and facility, and at times also incorporated staff’s maximum mileage, and county preferences. Further, we defined an urgency coefficient to give preference to facility demand with respect to need-by dates and prioritize facilities that are identified as critical by the stakeholders involved in responding to the COVID-19 pandemic.

The mixed-integer linear programming formulation of the matching problem is given as (see Table 2 for notation):

1 $$Min\sum_{j\in J}\sum_{i\in I}{x}_{ij}{w}_{ij}+\sum_{j\in J}\sum_{k\in K}{y}_{jk}\left(\frac{UD}{{u}_{j}}\right)$$

subject to:

2 $$\begin{array}{cc}\sum_{i\in I}{\alpha }_{ik}{x}_{ij}={D}_{jk}-{y}_{jk}& \forall j\in J, k\in K\end{array}$$

3 $$\begin{array}{cc}\sum_{j\in J}{x}_{ij}\le 1& \forall i\in I\end{array}$$

4 $$\begin{array}{cc}{\alpha }_{ik}{x}_{ij}\le \left(1-{\gamma }_{jk}\right)+{\theta }_{i}& \forall i\in I, j\in J, k\in K\end{array}$$

5 $$\begin{array}{cc}{x}_{ij}\le {v}_{i}+{p}_{j}& \forall i\in I, j\in J\end{array}$$

6 $$\begin{array}{cc}{\alpha }_{ik}{x}_{ij}\le {UL}_{jk}& \forall j\in J, k\in K\end{array}$$

7 $$\begin{array}{cc}1-{STS}_{i}{LTF}_{j}\ge {x}_{ij}& \forall i\in I, j\in J\end{array}$$

8 $$\begin{array}{cc}1-{STF}_{j}{LTS}_{i}\ge {x}_{ij}& \forall i\in I, j\in J\end{array}$$

9 $$\begin{array}{cc}{x}_{ij}\le {\pi }_{ij}& \forall i\in I, j\in J\end{array}$$

10 $$\begin{array}{cc}{x}_{ij}\in \left\{0\right.,\left.1\right\},{y}_{jk}\ge 0& \forall i\in I, j\in J, k\in K\end{array}$$

**Table 2:** Notation used for modeling the problem

Notation	Type of notation	Description
${\varvec{i}}$	Index	Staff index
${\varvec{j}}$	Index	Facility index
${\varvec{k}}$	Index	Position type index
${\varvec{I}}$	Set	Set of medical staff in the matching pool
${\varvec{J}}$	Set	Set of facilities in the matching pool
${\varvec{K}}$	Set	Set of positions (10 different types of positions)
${{\varvec{D}}}_{{\varvec{j}}{\varvec{k}}}$	Parameter	Demand of facility $j$ for position *k*
${\boldsymbol{\alpha }}_{{\varvec{i}}{\varvec{k}}}$	Parameter	Position indicator parameter, equals 1 if staff *i* is of position type *k*, and 0 otherwise
${{\varvec{\gamma}}}_{{\varvec{j}}{\varvec{k}}}$	Parameter	Demand license parameter, equals 1 if facility $j$ needs a licensed staff for position $k$, and 0 otherwise
${{\varvec{\theta}}}_{{\varvec{i}}}$	Parameter	Staff license parameter, equals 1 if staff $i$ has a valid license number, and 0 otherwise
${{\varvec{p}}}_{{\varvec{j}}}$	Parameter	Public transportation parameter, equals 1 if facility $j$ is near public transportation, and 0 otherwise
${{\varvec{v}}}_{{\varvec{i}}}$	Parameter	Vehicle ownership parameter, equals 1 if staff $i$ can travel by their own vehicle, and 0 otherwise
${{\varvec{S}}{\varvec{T}}{\varvec{S}}}_{{\varvec{i}}}$	Parameter	Short-term staff parameter, equals 1 if staff $i$ prefers short-term employment, and 0 otherwise
${{\varvec{L}}{\varvec{T}}{\varvec{S}}}_{{\varvec{i}}}$	Parameter	Long-term staff parameter, equals 1 if staff $i$ prefers long-term employment, and 0 otherwise
${{\varvec{L}}{\varvec{T}}{\varvec{F}}}_{{\varvec{j}}}$	Parameter	Long-term facility parameter, equals 1 if facility $j$ prefers long-term staff, and 0 otherwise
${{\varvec{S}}{\varvec{T}}{\varvec{F}}}_{{\varvec{j}}}$	Parameter	Short-term facility parameter, equals 1 if facility $j$ prefers short-term staff, and 0 otherwise
${{\varvec{\pi}}}_{{\varvec{i}}{\varvec{j}}}$	Parameter	Previously matched indicator parameter, equals 1 if staff $i$ is not previously matched to facility $j$, and 0 otherwise
${{\varvec{w}}}_{{\varvec{i}}{\varvec{j}}}$	Parameter	Weight coefficient associated with matching staff $i$ to facility $j$
${{\varvec{u}}}_{{\varvec{j}}}$	Parameter	Urgency coefficient for facility $j$, smaller value implies a higher urgency level
${\varvec{U}}{\varvec{D}}$	Parameter	The penalty associated with unmet demand
${{\varvec{U}}{\varvec{L}}}_{{\varvec{j}}{\varvec{k}}}$	Parameter	The upper limit on the maximum demand for position $k$ that can be satisfied for facility $j$
${{\varvec{x}}}_{{\varvec{i}}{\varvec{j}}}$	Decision Variable	Whether staff $i$ is matched to facility $j$ or not; ${{\varvec{x}}}_{{\varvec{i}}{\varvec{j}}\boldsymbol{ }}\in \{\mathrm{0,1}\}$
${{\varvec{y}}}_{{\varvec{j}}{\varvec{k}}}$	Decision Variable	Quantity of unsatisfied demand of facility $j$ for position type $k$

The objective function (1) minimizes the summation of total matching weights and unsatisfied demand penalty, which penalizes the unsatisfied demand based on its urgency level. Constraint (2) ensures that the total matched staff to a facility in each position type is equal to the total facility demand minus unmet demand. Constraint (3) establishes that each staff is matched with at most one facility. If a facility only needs a licensed staff for a position type, constraint (4) ensures that. Constraint (5) indicates that an individual is only matched with a facility if the staff can drive to work, or if the facility is near public transportation. Some facilities entered large demand quantities more frequently than other ones, and since only a limited number of staff were available, this behavior could prevent all facilities from receiving needed staff. Therefore, we added constraint (6), which limits the allocation to any one facility and helps distribute staff more evenly among facilities. Constraints (7) and (8) ensure that the employment preference (short-term/long-term) is the same between a matched staff and facility. Constraint (9) indicates that if a staff-facility pair is matched before, or if the facility did not hire a staff before, they do not get matched to each other again. Finally, constraints (10) are the binary and nonnegativity constraints for the decision variables.

### Solution methodology and output reports

To solve the developed matching model, we used CPLEX 12.8.0 on a computer with Intel Core i7-8550 CPU (1·8 GHz) and 8 GB of RAM with less than one minute running time for all instances. Then, we used the solution of the matching model to create facility matching reports and sent the reports to the facilities through the portal.

### Data sources

We used two datasets for the study, the CMS nursing home dataset [6] and the LTCF staffing portal generated dataset. First, the publicly available CMS nursing home dataset that NHs submit providing information on the number of cases and deaths among residents and staff at nursing homes for each county in Massachusetts.

Second, we used the Massachusetts COVID-19 LTCF staffing portal dataset. We constructed this dataset from the information generated through the portal including intake forms, matching results, and facility feedback. This dataset is only accessible to the portal operations team. The data contains 30 features (five categorical and 25 numerical variables) including facility feedback and staff and facility characteristics such as their zip codes, staff shift and work preferences, years of experience, and facility type and number of demand records. Each row of this dataset refers to a unique pair of facility-staff match. Furthermore, we cleaned the dataset by excluding 24 facilities that did not use the portal as intended.

### Statistical analysis

One of the most challenging aspects of operating the portal was the lack of feedback entries from the LTCFs on their decisions about matched staff. LTCFs’ decisions indicate whether the facility is intending to hire the matched staff or not. With the limited feedback entries, the staff who had been previously matched was kept in the eligible staff pool and rematched to other facilities multiple times. This created several undesirable operational issues in the system, such as multiple facilities competing for the same staff or undesirable staff not being identified and taken out of the pool. Therefore, we analyzed the LTCFs’ feedback data entered through the portal to better understand which facility and staff features impact whether a facility provided feedback on matched staff.

We applied different methods for feature selection to create a robust logistic regression model and reduce collinearity between variables. We mainly used Pearson correlation analysis to find highly correlated variables and eliminate a subset of them [28]. Finally, we selected thirteen features, including type of facility (including nursing home, assisted living center, and others, reference group: others), number of times a facility has been prioritized due to low staffing levels (identified by the the MA COVID-19 taskforce), and whole facility testing (i.e., scheduled mandatory COVID-19 testing for all residents and staff at an LTCF), number of demand records entered by a facility, work preferences of staff (including per diem, long term, and either, reference group: either), shift preferences of staff (6 features, including weekday morning, weekday afternoon-evening, weekday overnight, weekend morning, weekend afternoon-evening, and weekend overnight), distance from staff to the matched facility, and staff’s years of experience.

The outcome variable in the logistic regression model is a binary variable showing whether a facility has provided feedback for the matched staff or not, which we obtained from the Massachusetts COVID-19 LTCF staffing portal dataset.

## Results

We analyze the outcomes of the matching process to determine whether the developed matching framework was successful in responding to facilities' demand and providing job opportunities for medical staff. We also hope to learn how the framework can be improved in terms of providing matches with higher hiring rates and determine the important characteristics that resulted in facilities providing feedback on hiring decisions.

**Fig. 5:**
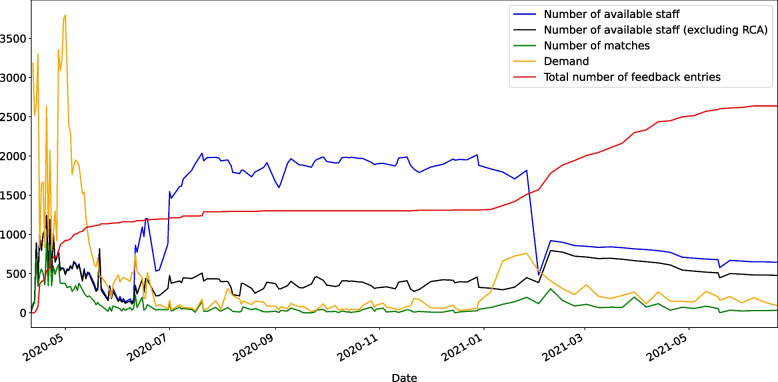
Overtime total eligible demand and available staff, number of matches, and total number of feedback entries

The matching portal was operational between April 2020 and June 2021, and was updated several times based on the changing state policies and needs during its lifetime. The matching framework was used roughly 150 times during its operational time, 19,084 unique matches were made, and facilities provided feedback for 2,542 matches. Figure 5 represents the number of available staff including RCAs (blue line) and excluding them (black line) along with the eligible demand in the matching pool (green line), the number of matches (orange line), and the feedback level that the facilities provided (red line) for each round of matching over time. The figure demonstrates how the matching framework has been responsive to the demand based on supply availability on each matching date. Moreover, this figure shows that the facility feedback rate significantly increased after an improved version of the portal (portal 3.0) went live on January, 2021. This version of the portal provided a more convenient way for facilities to provide feedback on matches.

**Fig. 6:**
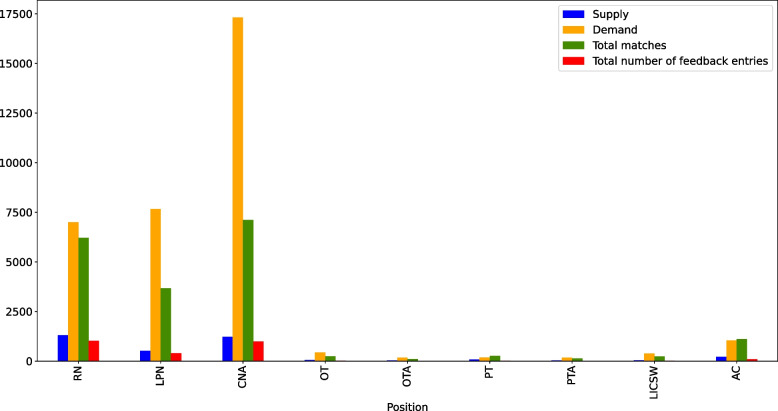
Number of supply (registered staff), demand, number of matches and total number of feedback entries for each position

For understanding response of the framework for each position, Fig. 6 shows the distributions of the number of staff, demand quantity, number of matches, and number of feedback entries for nine different positions (excluding RCAs). This figure indicates that, except for PT and AC positions, the portal could not match the facility demand due to staff supply levels. By comparing blue and orange bars, this figure indicates the severity of staff shortages in LTCFs, specially for the three nursing positions RN, CNA, and LPN. Further, this figure shows that demand exceeds number of available staff for all positions and most of the demand entries and feedback entries are for the three nursing positions RN, CNA, and LPN.

**Fig. 7:**
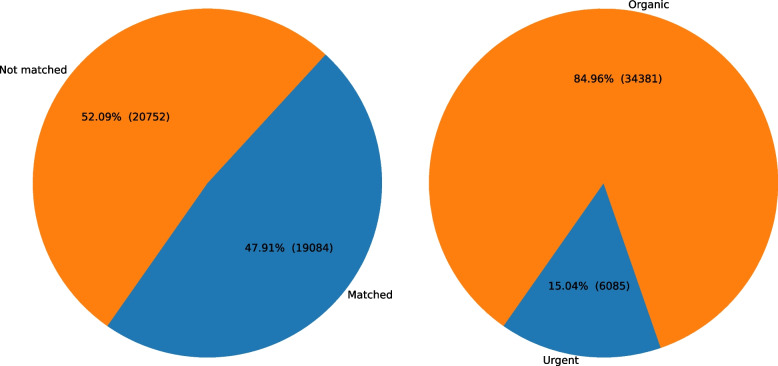
Distribution of facility demand satisfaction (left) and distribution of organic and urgent demand (right)

In addition to the number of matches and facilities’ feedback entries for each position, shown in Figs. 6 and 7 indicates the percentage of demand satisfaction for facilities (Fig. 7 left), and organic and urgent demand quantities (Fig. 7 right) as other metrics. Organic demand refers to the demand that the facilities entered in the portal. On the other hand, urgent demand was created for the facilities that were prioritized for matching by the stakeholders involved in responding to the COVID-19 pandemic due to low staffing levels or expected shortages from all facility testing. Based on Fig. 7, 47.91% (19,084 out of 39,836) of the facility demand entries were matched to and 15.04% (6,085 out of 40,466) of the total demand were urgent.

**Fig. 8:**
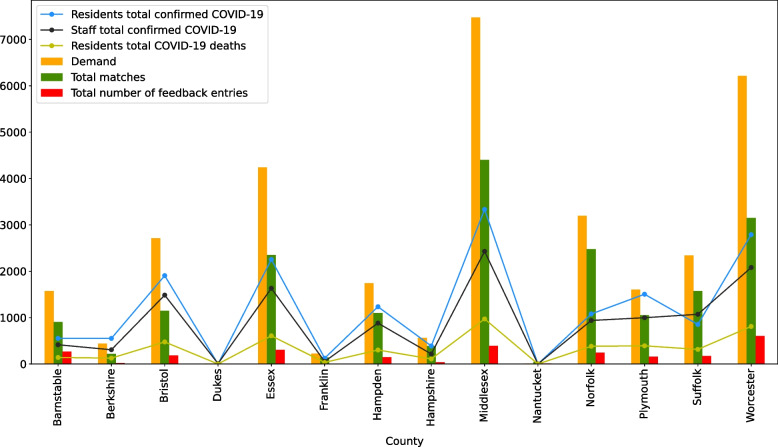
Number of total resident confirmed cases, total resident COVID-19 deaths, staff total confirmed cases, demand, total matches, and number of feedback entries for all LTCFs in each county in Massachusetts

Using the CMS NH dataset and the LTCF portal generated dataset, we depicted in Fig. 8 the demand, total matches, and the number of feedback entries with the number of resident and staff COVID-19 cases and the number of total resident deaths in each county in Massachusetts.^5^ From this figure, we note that counties with higher number of cases among residents and staff and number of deaths among residents entered more demand in the portal and as a result, received more matches. However, portal activity was at a higher level in Middlesex and Worcester county facilities in proportion to Covid cases and resident deaths.

On average, facilities provided feedback for 13.32% (2,542 out of 19,084) of the matched staff with 478 (2.50%) feedback showing facilities did not hire matched staff and 2,064 (10.82%) feedback showing facilities intend to hire matched staff.

**Fig. 9:**
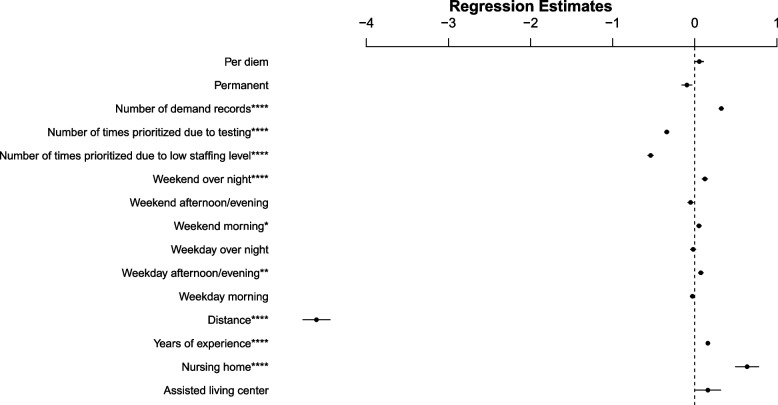
Standardized coefficients of variables NOTES Estimated standardized coefficients with 95% CI (confidence interval) for each variable in the logistic regression model. Reference groups are Either (work preference), and Other (types of facilities). The outcome is a binary variable indicating whether facilities provide response for a match (equals 1) or not (equals 0). *p < 0·10, **p < 0·05, ***p < 0.01, ****p < 0.0001

We used variables’ standardized coefficients to examine the association of variables with facilities entering feedback on the matched staff. Table 3 presents the results and associations of variables with facility feedback entry. We conclude that NH and LTCFs with more demand entries were more likely to provide feedback. Moreover, staff’s years of experience, shift preferences including weekday afternoon-evening, weekend morning, and weekend overnight are associated with facilities providing feedback on the matched staff. In addition, number of times LTCFs are prioritized due to testing or low staffing levels are associated with LTCFs that did not provide feedback on the matched staff. Finally, the distance between the staff and the facility that they were matched to is associated with a higher likelihood of a facility providing feedback on matched staff. Figure 9 indicates the standardized coefficients of variables with a line showing 95% confidence interval (CI).

**Table 3:** Associations of variables with facility feedback on matched staff

	**Variable**	**Coefficient**	**Standard error**	***P*** **-value**	**95% CI**
**Facility type**	Assisted living center	0.160	0.156	0.306	(-0.146, 0.466)
	Nursing home	0.638	0.141	< 0.0001****	(0.362, 0.914)
	Others	Ref	-	-	-
	Years of experience	0.160	0.022	< 0.0001****	(0.117, 0.202)
	Distance	-4.608	0.166	< 0.0001****	(-4.932, -4.283)
**Shift preferences**	Weekday morning	-0.028	0.027	0.287	(-0.081, 0.024)
	Weekday afternoon/evening	0.074	0.029	0.011**	(0.017, 0.131)
	Weekday over night	-0.019	0.032	0.540	(-0.082, 0.042)
	Weekend morning	0.051	0.029	0.073*	(-0.005, 0.107)
	Weekend afternoon/evening	-0.050	0.032	0.120	(-0.114, 0.013)
	Weekend overnight	0.124	0.033	0.0002***	(0.058, 0.189)
**Prioritization**	Number of times prioritized due to low staffing levels	-0.537	0.032	< 0.0001****	(-0.601, -0.474)
	Number of time prioritized due to testing	-0.341	0.027	< 0.0001****	(-0.393, -0.288)
	Number of demand records	0.324	0.027	< 0.0001****	(0.271, 0.376)
**Work preference**	Permanent	-0.096	0.061	0.118	(-0.216, 0.024)
	Per diem	0.056	0.051	0.272	(-0.044, 0.156)
	Either	Ref	-	-	-

CI is confidence interval. Reference groups are Either (work preference), and Other (types of facilities). The outcome is a binary variable indicating whether facilities provide response for a match (equals 1) or not (equals 0). This logistic regression model was applied to 19,084 matches. *p < 0·10, **p < 0·05, ***p < 0.01, ****p < 0.0001

## Discussion

During the COVID-19 outbreak, LTCFs faced severe staff shortage issues. In this study, in collaboration with the EOEA, we developed a portal and a matching framework to alleviate staffing problems in LTCFs in Massachusetts. The developed matching framework facilitates staff allocation by considering restrictions of facilities and staff. The underlying matching model was adopted to the specific and changing situation on the ground and considered a variety of criteria for matching, such as the distance between facilities and staff while also incorporating restrictions due to licensing and transportation needs. We used the matching framework dynamically by updating the matching pools and adjusting daily facility prioritizations. Furthermore, the matching framework went through several updates and modifications. New features that were added to the framework overtime included improved data entry portals for both the facilities and staff and methodology for updating facility and staff pools to achieve more effective matches by removing excessive facility demand and inactive staff.

Despite continuous refinement and improvements on the matching framework, this study and the framework are subject to several limitations, including: (i) Due to work overload, LTCF staff was not always able to take action and provide feedback on the matches provided to a facility. While the portal user interface was updated several times and several follow up calls were made to facilities, the issue persisted for some. (ii) A subset of the mathematical model parameters such as matching weights and shortage penalty (i.e., a parameter that enforces the matching model to allocate available staff to LTCFs and always match an available staff to a facility if feasible) are estimated based on expert opinion and subjective; (iii) Several potentially important considerations, such as shift preferences, were not included as criteria in the matching process but only reported to the facilities as information along with their matched staff due to concerns regarding how to elicit preference information from the facilities without imposing too high of a data entry burden. We believe including such criteria will increase the quality of the matches; (iv) The matching framework only considered the current demand during each run. This led to matching all available staff to the current demand if possible, ignoring the potential for demand surges or critical demand that could arise in the future. An enhanced framework could be established by forecasting future demand based on evolving trends of the public emergency and reserving some supply for covering this future demand. (v) It was observed that a subset of the facilities inflated their demand in order to increase their matches. While the current framework tried to prevent hoarding by implementing simple limits such as those based on bed numbers, a more sophisticated process could be designed to disincentivize this behavior and recognize and adjust allocations accordingly.

In conclusion, we developed a centralized matching framework that can be an efficient tool for responding to staff shortages during public health emergencies. We further analyzed the impact of using the matching framework on staffing level of NHs in a subsequent paper [29]. Moreover, the analytical results imply that NHs that were using the portal regularly were more likely to provide feedback. Finally, staff that were matched to a close facility, had work experience, and were willing to work in the afternoons or later were likely to drive hiring feedback from the facility.

## Data Availability

One of the datasets analyzed during the current study is available in the Centers for Medicare and Medicaid Services repository, [https://data.cms.gov/Special-Programs-Initiatives-COVID-19-Nursing-Home/COVID-19-Nursing-Home-Dataset/s2uc-8wxp/data]. The other dataset analyzed in this study is not publicly available due to privacy of facilities and staff but are available from the corresponding author on reasonable request.

## Footnotes

1
https://www.mass.gov/doc/dcs-info-34-105f-1000-hiring-bonus-faq/download.

2 RCA position was created especially to offset the increasing demand for CNAs. While the LTCFs never entered much demand for RCAs compared to other positions, the portal matched RCAs to CNA positions whenever there were insufficient CNAs to meet the demand. We excluded this position from our analysis throughout the article.
